# Clinical Lectures on Diseases of the Eye

**Published:** 1865-06

**Authors:** E. L. Holmes

**Affiliations:** Chicago, Lecturer on Diseases of the Eye and Ear in Rush Medical College, and Surgeon of the Chicago Charitable Eye and Ear Infirmary


### CLINICAL LECTURES ON DISEASES OF THE EYE.

By E. L. HOLMES, M. D., of Chicago,
*Lecturer on Diseases of the Eye and Ear in Rush Medical College, and Surgeon of the Chicago Charitable Eye and Ear Infirmary.*

### THE SCLEROTICA, OR SCLERA.

*Gentlemen:*—The case of Hydrophthalmos and of partial staphyloma of the sclerotic, which I have presented to your notice within the past fortnight, are examples of the diseases of which I propose to speak to you to-day.

The sclerotic is the dense, white membrane, with scarcely any elasticity, which gives firmness to the walls of the globe. It varies in thickness from two-fifths to one-half a line, being usually thicker at its posterior than its anterior half. In old age it becomes harder, and at the same time thinner, than in youth. It is composed of several ill-defined layers of white tissue, the fibres of which are so interlaced with each other that the layers can be separated with difficulty. There are also a few fibres of elastic tissue, and also a large number of cells mingled with the fibrous tissue. Anteriorly, the structure of the sclerotic passes abruptly into that of the cornea. Posteriorly, the external layers of the sclerotic continue backwards as a covering of the optic nerve, which may also be considered as a continuation of the dura mater of the brain, through the optic foramen. The opening through the sclerotic for the optic nerve is not a simple aperture, but is a very large number of exceedingly minute openings, formed by delicate fibres of the sclerotic, passing across each other, like the wires of a seive, through each of the meshes of which passes a single fibrile of the optic nerve. This seive-like structure is called the lamina cribrosa. Near the line of demarkation between the cornea and sclerotic is a minute canal in the latter, passing like a ring around the cornea, called the canal or sinus of Schlemm, which seems to receive a portion of the blood from the choroid and ciliary processes. The sclerotic is pierced by a large number of minute vessels and nerves, especially in the region of the optic nerve and the ciliary processes, which supply the intra-ocular tissues. The sclerotic itself possesses scarcely any trace of nerves. The vessels are exceedingly minute and distributed only sparsely through it.

The inner surface of the sclerotic is quite smooth, and loosely attached to the choroid, except near the optic nerve and cornea. The external surface is covered with a layer of very loose areola tissue, the outer portion of which is quite firm. This layer forms a kind of capsule or sub-mucous tissue and is called the epi-sclerotic membrane. Its anterior hemisphere is sometimes called the capsule of Thenon; its posterior, the capsule of Bonnet. The epi-sclera is richly supplied with vessels, which are usually the first congested in inflammations of the iris and choroid.

The diseases of the sclerotic are, almost without exception, secondary, dependent, in the form of inflammation, upon the injuries or diseases of the cornea, iris, or choroid, and possibly of the conjunctiva. Some authors have doubted whether idiopathic inflammation of the sclerotic ever occurs. There is reason, however, to believe that in rare instances, inflammation may attack the sclerotic primarily.

However caused, the inflammatory action presents the following phenomena: The cells of the sclerotic become enlarged, their granular contents form into new cells, which from their increase of numbers, produce thickening of tissue, and if the process continues, total or partial destruction of intercellular tissue. In the one case there may be a slough, or in the other there may be such a softening and weakening of the walls of the globe, as to render them insufficient to withstand even the normal pressure of the intra-ocular fluids. In the last case a staphyloma of the sclerotic is the result.

By far the most common cause of these abnormal conditions is choroiditis. Hence the disease is often termed sclero-choroiditis. According as the disease is confined to a portion of the globe or extends to the whole, it is called partial or total staphyloma. In my own experience all cases of staphyloma of the sclerotic have been confined to patients below middle age. In more advanced years, the sclerotic becomes harder and seems better able to resist the influence of choroidal inflammation.

In total staphyloma of the sclerotic, or as it is often called, hydrophtalmos, there is often a softening of the whole sclerotic and cornea, and consequent dilatation of these membranes to such an extent, that the globe becomes enormously enlarged. The cornea in such cases is exceedingly thin and dilated to even double its normal size. The iris is much attenuated and forced forward in contact with the cornea, or becomes atrophied. The ciliary processes also lose their normal structure, and sometimes almost wholly disappear.

The lens will be found, in some cases, in a calcareous condition, or as a shapeless mass, attached to the inner surface of the cornea. In other cases it is almost completely destroyed. The vitreous humor suffers in structure, being either partially or wholly liquified. The retina presents different appearances in different cases, sometimes almost wholly disappearing, and sometimes remaining as a thin membrane, without blood vessels, attached to the optic nerve and floating in the abnormal fluids of the eye. The papilla is usually cupped, as described in the last lecture; it is, however, occasionally found upon examination, after extirpation of the globe, as a plain disc.

Partial staphyloma of the sclerotic is a projection of a portion of this membrane, generally produced either by injury, or by circumscribed inflammation of the choroid. It may appear as quite a large, ill-defined elevation on one or two sides of the eye, or as a dark-colored tumor of the size of a pea, a few lines posterior to the cornea. This latter form often depends upon inflammation of the ciliary processes, and is sometimes so extensive that the cornea is completely surrounded by small, dark-colored tumors.

As can be readily imagined, vision in nearly all cases of sclerotic disease is seriously implicated. Although the disease may sometimes remain stationary for a long period, it too often progresses, frequently with great pain and symptoms of sympathetic irritation of the other eye. The result may be atrophy, either with or without evacuation of the contents of the globe, through rupture of the walls of the staphyloma.

The principal points to be observed in treatment, are the following: In the early stages, iridectomy may prove advantageous, as tending to reduce choroidal inflammation, and remove intra-ocular pressure. In more advanced stages, repeated paracentesis, or removal of the staphyloma, as in staphyloma of the cornea, have been recommended by ophthalmic surgeons. Except under very favorable circumstances, however, either of these operations is followed by more or less intense inflammation, and a long period elapses before the atrophied eye is without irritation, and tenderndess on pressure. Some distinguished oculists recommend the total extirpation of the globe. I am confident that in a large proportion of cases, the removal of the diseased globe will give much more speedy relief, with less danger of sympathetic irritation in the other eye, than the other modes of treatment which have been proposed.

Another form of disease of the sclerotic is termed staphyloma posticum, usually marked by a circumscribed projection in the sclerotic external to the optic nerve. With this condition there is a tendency to elongation of the antero posterior diametre of the eye. This elongated form of the eye can sometimes be observed by simple inspection, when the globe is rotated towards the inner angle.

The disease is recognized in an examination with the ophthalmoscope by the presence of a crescent shaped appearance around the temporal side of the papilla of the optic nerve. Almost the only subjective symptom in the early stages of the affection is myopia, its degrees depending upon the severity of the case. In the earlier stages this symptom may possibly be absent, where the power of accommodation for vision at different distances is very great. The near sightedness is caused by the increase of distance between the lens and retina, in consequence of the increase of the diametre of the globe adjust described.

The cause of the disease is obscure. It seems generally to depend upon some defect in the fœtal development of the posterior part of the sclerotic. It is not now considered as the result of inflammatory action, since there are none of the symptoms of inflammation, vision often being perfect in every respect except for distant objects. In conjunction with the congenital tendency to the disease, undue action of the recti muscles, as in fixing the eyes upon near objects, seems to act as an exciting cause.

Comparatively little can be accomplished in the curative treatment. Usually the most that can be promised, is to arrest the progress of the disease. To this end, everything, which produces congestion, as fatigue of the eyes, especially when used long at a time, should be avoided. If at any time there is an abnormal degree of intra-occular pressure, which would tend to increase the staphyloma, iridectomy or division of the ciliary muscle may be required. It should be remembered that in many cases, in spite of all treatment, the prognosis is very grave, the disease terminating in excessive myopia, and finally total loss of sight.

